# Prediction of Protein-Protein Interaction Strength Using Domain Features with Supervised Regression

**DOI:** 10.1155/2014/240673

**Published:** 2014-06-24

**Authors:** Mayumi Kamada, Yusuke Sakuma, Morihiro Hayashida, Tatsuya Akutsu

**Affiliations:** ^1^Department of Biosciences and Informatics, Keio University, 3-14-1 Hiyoshi, Kohoku-ku, Yokohama 223-8522, Japan; ^2^Japan Ichiba Section Development Unit, Rakuten Inc., 4-12-3 Higashi-shinagawa, Shinagawa-ku, Tokyo 140-0002, Japan; ^3^Bioinformatics Center, Institute for Chemical Research, Kyoto University, Gokasho, Uji, Kyoto 611-0011, Japan

## Abstract

Proteins in living organisms express various important functions by interacting with other proteins and molecules. Therefore, many efforts have been made to investigate and predict protein-protein interactions (PPIs). Analysis of strengths of PPIs is also important because such strengths are involved in functionality of proteins. In this paper, we propose several feature space mappings from protein pairs using protein domain information to predict strengths of PPIs. Moreover, we perform computational experiments employing two machine learning methods, support vector regression (SVR) and relevance vector machine (RVM), for dataset obtained from biological experiments. The prediction results showed that both SVR and RVM with our proposed features outperformed the best existing method.

## 1. Introduction

In cellular systems, proteins perform their functions by interacting with other proteins and molecules, and protein-protein interactions (PPIs) play various important roles. Therefore, revealing PPIs is a key to understanding biological systems, and many investigations and analyses have been done. In addition, a variety of computational methods to predict and analyze PPIs have been developed, for example, methods for predicting PPI pairs using only sequences information [1–5], for predicting amino acid residues contributing to PPIs [6–8], and for assessing PPI reliability in PPI networks [9, 10]. As well as studies of PPIs, analyses of strengths of PPIs are important because such strengths are involved in functionality of proteins. In terms of transcription factor complexes, if a constituent protein has a weak binding affinity, target genes may not be transcribed depending on intracellular circumstance. For example, it is known that multi-subunit complex NuA3 in* Saccharomyces Cerevisiae* consists of five proteins, Sas3, Nto1, Yng1, Eaf6, and Taf30, acetylates lysine 14 of histone H3, and activates gene transcription. However, only Yng1 and Nto1 are found solely in the complex, and interaction strengths between each component protein are thought to be different and transient. Hence, Byrum et al. proposed a biological methodology for identifying stable and transient protein interactions recently [11].

Although many biological experiments have been conducted for investigating PPIs [12, 13], strengths of PPIs have not been always provided. Ito et al. conducted large-scale yeast two-hybrid experiments for whole yeast proteins. In their experiments, yeast two-hybrid experiments were conducted for each protein pair multiple times, the number of experiments that observe interactions, or the number of interaction sequence tags (ISTs), was counted. Consequently, they decided that protein pairs having three or more ISTs should interact and reported interacting protein pairs.

The ratio of the number of ISTs to the total number of experiments for a protein pair can be regarded as the interaction strength between their proteins. On the basis of this consideration, several prediction methods for strengths of PPIs have been developed. LPNM [14] is a linear programming-based method; ASNM [15] is a modified method from the association method [16] for predicting PPIs. Chen et al. proposed association probabilistic method (APM) [17], which is the best existing method for predicting strengths of PPIs as far as we know.

These methods are based on a probabilistic model of PPIs and make use of protein domain information. Domains are known as structural and functional units in proteins and well-conserved regions in protein sequences. The information of domains is stored in several databases such as Pfam [18] and InterPro [19]. The same domain can be identified in several different proteins. In these prediction methods, interaction strengths between domains are estimated from known interaction strengths between proteins, and interaction strengths for target protein pairs are predicted from estimated strengths of domain-domain interactions (DDIs).

On the other hand, Xia et al. proposed a feature-based method using neural network with features based on constituent domains of proteins [20], and they compared their method with the association method and the expectation-maximization method [21]. For the feature-based prediction of PPI strengths, we also utilize domain information and propose several feature space mappings from protein pairs. We use supervised regression and perform threefold cross validation for dataset obtained from biological experiments. This paper augments the preliminary work presented in conference proceedings [22]. Specifically, major augmentations of this paper and differences from the preliminary conference version are summarized as follows.

- We employ two supervised regression methods: support vector regression (SVR) and relevance vector machine (RVM). Note that we used only SVR with the polynomial kernel in the preliminary version [22].
- The Laplacian kernel is used as the kernel function for SVR and RVM, and kernel parameters are selected via fivefold cross validation.
- We prepare the dataset from WI-PHI dataset [23] with high reliability.

The computational experiments showed that the average root mean square error (RMSE) by our proposed method was smaller than that by the best existing method, APM [17].

## 2. Materials and Methods

In this section, we briefly review a probabilistic model and related methods, and propose several feature space mappings using domain information.

### 2.1. Probabilistic Model of PPIs Based on DDIs

**Figure 1:**
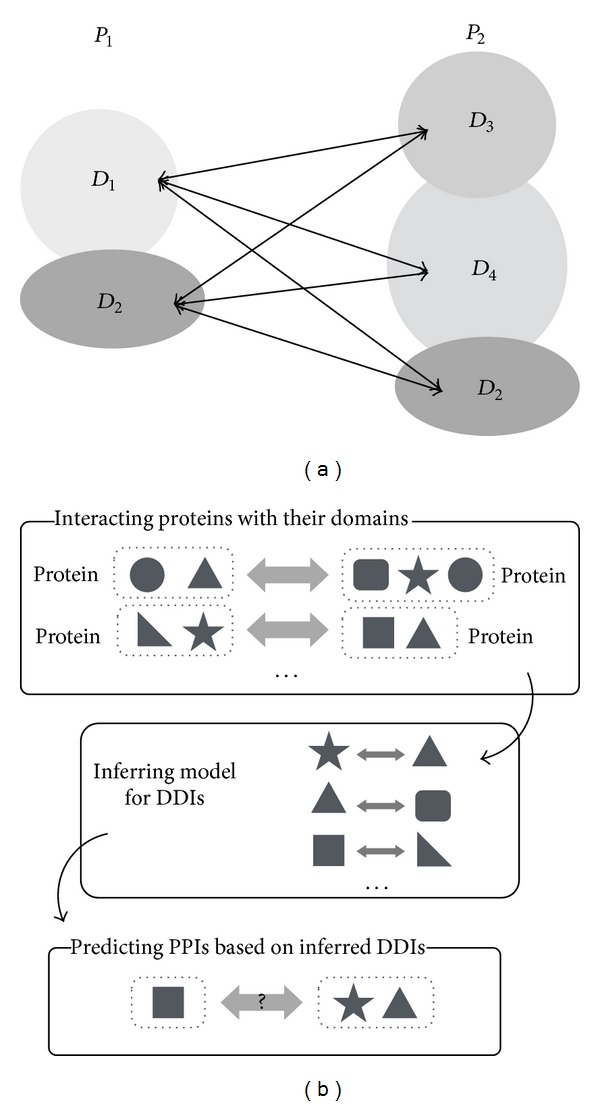
(a) Illustration of protein-protein interactions (PPIs) model based on domain-domain interactions (DDIs). (b) Schematic overview of PPIs prediction based on DDIs.

There are some computational prediction methods for PPI strengths, and they are based on the probabilistic model of PPIs proposed by Deng et al. [21]. This model utilizes DDIs and assumes that two proteins interact with each other if and only if at least one pair of the domains contained in the respective proteins interacts.  Figure 1(a) illustrates an example of this interaction model. In this example, there are two proteins *P*_1_ and *P*_2_, which consist of domains *D*_1_, *D*_2_ and domains *D*_2_, *D*_3_, *D*_4_, respectively. According to Deng's model, if *P*_1_ and *P*_2_ interact, at least one pair among (*D*_1_, *D*_2_), (*D*_1_, *D*_3_), (*D*_1_, *D*_4_), (*D*_2_, *D*_2_), (*D*_2_, *D*_3_), and (*D*_2_, *D*_4_) interacts. Conversely, if a pair, for instance, (*D*_2_, *D*_4_), interacts, *P*_1_ and *P*_2_ interact. From the assumption of this model, we can derive the following simple probability that two proteins *P*_*i*_ and *P*_*j*_ interact with each other:

(1) $$\begin{array}{c} \text{Pr}\left(P_{ij}=1\right)=1-\underset{D_{m}\in P_{i},D_{n}\in P_{j}}{\prod }\left(1-\text{Pr}\left(D_{mn}=1\right)\right), \end{array}$$

where *P*_*ij*_ = 1 indicates the event that proteins *P*_*i*_ and *P*_*j*_ interact (otherwise, *P*_*ij*_ = 0), *D*_*mn*_ = 1 indicates the event that domains *D*_*m*_ and *D*_*n*_ interact (otherwise, *D*_*mn*_ = 0), and *P*_*i*_ and *P*_*j*_ also represent the sets of domains contained in *P*_*i*_ and *P*_*j*_, respectively. Deng et al. applied the EM (expectation maximization) algorithm to the problem of maximizing log-likelihood functions, the estimated probabilities that two domains interact, Pr⁡(*D*_*mn*_ = 1), and proposed a method for predicting PPIs using the estimated probabilities of DDIs [21]. Actually, they calculated Pr⁡(*P*_*ij*_ = 1) using (1) and determined whether or not *P*_*i*_ and *P*_*j*_ interact by introducing a threshold *θ*; that is, *P*_*i*_ and *P*_*j*_ interact if Pr⁡(*P*_*ij*_ = 1) ≥ *θ*; otherwise, the proteins do not interact.

As Deng's method, typical PPIs prediction methods based on domains have the following two steps. First the interaction between domains contained in interacting proteins is inferred from existing protein interaction data. And then, an interaction between new protein pairs is predicted on the basis of the inferred domain interactions using a certain model.  Figure 1(b) illustrates the flow of this type of PPIs prediction. Since interacting sites may not be always included in some known domain region, it can cause the decrease of prediction accuracy in this framework.

### 2.2. Association Method: Inferring DDI from PPI Data

As described previously, probability of PPIs could be predicted based on probabilities of DDIs. In this subsection, we will briefly review related methods to estimate a probability of interaction for domain pair.

### 2.3. Association Method

Let *P* be a set of protein pairs that have been observed to interact or not. The association method [16] gives the following simple score for two domains *D*_*m*_ and *D*_*n*_ using proteins that include the following domains:

(2) ASSOC(Dm,Dn) =|{(Pi,Pj)∈P ∣ Dm∈Pi,Dn∈Pj,Pij=1}||{(Pi,Pj)∈P ∣ Dm∈Pi,Dn∈Pj}|,

where |*S*| indicates the number of elements contained in the set *S*. This score represents the ratio of the number of interacting protein pairs including *D*_*m*_ and *D*_*n*_ to the total number of protein pairs including *D*_*m*_ and *D*_*n*_. Hence, it can be considered as the probability that *D*_*m*_ and *D*_*n*_ interact.

### 2.4. Association Method for Numerical Interaction Data (ASNM)

Originally the association method has been designed for inferring binary protein interactions. To predict numerical interactions such as interaction strengths, Hayashida et al. proposed the association method for numerical interaction (ASNM) by the modification of the original association method [15]. This method takes strengths of PPIs as input data. Let *ρ*_*ij*_ represent the interaction strength between *P*_*i*_ and *P*_*j*_, and we suppose that *ρ*_*ij*_ is defined for all (*P*_*i*_, *P*_*j*_) ∈ *P*. Then, the ASNM score for domains *D*_*m*_ and *D*_*n*_ is defined as the average strength over protein pairs including *D*_*m*_ and *D*_*n*_ by

(3) $$\begin{array}{c} \text{ASNM}\left(D_{m},D_{n}\right)=\frac{\sum_{\left\{(P_{i},P_{j})\in P\;|\;D_{m}\in P_{i},D_{n}\in P_{j}\right\}}\rho_{ij}}{\left|\left\{\left(P_{i},P_{j}\right)\in P\;|\;D_{m}\in P_{i},D_{n}\in P_{j}\right\}\right|}. \end{array}$$

If *ρ*_*ij*_ always takes only 0 or 1, ASNM(*D*_*m*_, *D*_*n*_) becomes ASSOC(*D*_*m*_, *D*_*n*_).

### 2.5. Association Probabilistic Method (APM)

Although ASNM is a simple average of strengths of PPIs, Chen et al. proposed the association probabilistic method (APM) by replacing the strength with an improved strength [17]. It is based on the idea that the contribution of one domain pair to the strength of PPI should vary depending on the number of domain pairs included in a protein pair. They assumed that the interaction probability of each domain pair is equivalent in a protein pair, and transformed (1) as follows:

(4) $$\begin{array}{c} \text{Pr}\left(D_{mn}=1\right)=1-{\left(1-\text{Pr}\left(P_{ij}=1\right)\right)}^{1/|P_{i}||P_{j}|}. \end{array}$$

Thus, by substituting the numerator of ASNM, APM is defined by

(5) APM(Dm,Dn) =∑{(Pi,Pj)∈P ∣ Dm∈Pi,Dn∈Pj}(1−(1−ρij)1/|Pi||Pj|)|{(Pi,Pj)∈P ∣ Dm∈Pi,Dn∈Pj}|.

They conducted some computational experiments, and reported that APM outperforms existing prediction methods such as ASNM and LPNM.

### 2.6. Proposed Feature Space Mappings from Protein Pairs

The association methods including ASNM and APM are based on the probabilistic model of PPIs defined by (1), and infer strengths of PPIs from estimated DDIs using given frequency of interactions or interaction strengths of protein pairs. On the other hand, we can also infer PPI strengths utilizing features obtained from given information such as sequence and structure of proteins with machine learning methods. Xia et al. proposed a method to infer strengths of PPIs using artificial neural network with features from constituent domains of proteins [20]. In this paper, for predicting strengths of PPIs, we propose several feature space mappings from protein pairs making use of domain information.

### 2.7. Feature Based on Number of Domains (DN)

As described above, constituent domains information is useful for inferring PPIs and also can be used as a representation of each protein. Actually, Xia et al. represented each protein by binary numbers indicating whether a protein has a domain or not based on the information of constituent domains, and used them with the artificial neural network to predict PPI strengths [20]. Here, it can be considered that the probability that two proteins interact increases with a larger number of domains included in the proteins. Therefore, in this paper, we propose a feature space mapping based on the number of constituent domains (called DN) from two proteins. The feature vector of DN for two proteins *P*_*i*_ and *P*_*j*_ is defined by

(6) $$\begin{array}{c} f_{ij}^{\left(m\right)}=M\left(D_{m},P_{i}\right)\;\text{for}\;\;D_{m}\in P_{i},\\ f_{ij}^{\left(T+n\right)}=M\left(D_{n},P_{j}\right)\;\text{for}\;\;D_{n}\in P_{j},\\ f_{ij}^{\left(l\right)}=0\;\text{for}\;\;D_{l}\notin P_{i}\cup P_{j}, \end{array}$$

where *T* indicates the total number of domains over all proteins and *M*(*D*_*m*_, *P*_*i*_) indicates the number of domains identified as *D*_*m*_ in protein *P*_*i*_.

### 2.8. Feature by Restriction of Spectrum Kernel to Domain Region (SPD)

**Figure 2:**
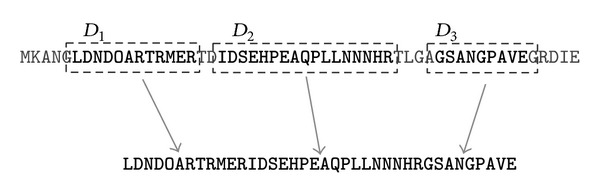
Illustration of restricting an amino acid sequence to which the spectrum kernel is applied to the domain regions.

DN is based only on the number of constituent domains of each protein, while amino acid sequences of domains are also considered useful for inferring strength of PPI. Therefore, we propose a feature space mapping by restricting the application of the spectrum kernel [24] to domain regions (called SPD). Let *A* be the set of 21 alphabets representing 20 types of amino acids and others. Although we used the set of 20 alphabets to express 20 types of amino acids in the preliminary conference version [22], we add one alphabet to take the ambiguous amino acids such as X into consideration. Then, *A* ^*k*^ (*k* ≥ 1) means the set of all strings with length *k* generated from *A*. The *k*-spectrum kernel for sequences *x* and *y* is defined by

(7) $$\begin{array}{c} K_{k}\left(x,y\right)=\langle \Phi_{k}\left(x\right),\Phi_{k}\left(y\right)\rangle , \end{array}$$

where Φ_*k*_(*x*) = (*ϕ*_*s*_(*x*))_*s*∈*A*^*k*^_ and *ϕ*_*s*_(*x*) indicates the number of times that *s* occurs in *x*. To make use of domain information, we restrict an amino acid sequence to which the *k*-spectrum kernel is applied to the domain regions.  Figure 2 illustrates the restriction. In this example, the protein consists of domains *D*_1_, *D*_2_, *D*_3_, and each domain region is surrounded by a square. Then, the subsequence in each domain is extracted, and all the subsequences in the protein are concatenated in the same order as domains. We apply the *k*-spectrum kernel to the concatenated sequence. Let *ϕ*_*s*_^(*r*)^(*x*) be the number of times that string *s* occurs in the sequence restricted to the domain regions in protein *x* in the above manner. The feature vector of SPD for proteins *P*_*i*_ and *P*_*j*_ is defined by

(8) $$\begin{array}{c} f_{ij}^{l}=\phi_{s_{l}}^{\left(r\right)}\left(P_{i}\right)\;\text{for}\;\;s_{l}\in A^{k},\\ f_{ij}^{\left(21^{k}+l\right)}=\phi_{s_{l}}^{\left(r\right)}\left(P_{j}\right)\;\text{for}\;\;s_{l}\in A^{k}. \end{array}$$

It should be noted that *ϕ*_*s*_^(*r*)^ for proteins having the same composition of domains can vary depending on the amino acid sequences of their proteins. That is, even if *P*_*i*_ and *P*_*j*_ have the same compositions as *P*_*k*_ and *P*_*l*_, respectively, and the feature vector of DN for *P*_*i*_ and *P*_*j*_ is the same as that for *P*_*k*_ and *P*_*l*_, then the feature vector of SPD for *P*_*i*_ and *P*_*j*_ can be different from that for *P*_*k*_ and *P*_*l*_.

### 2.9. Support Vector Regression (SVR)

To predict strengths of PPIs, we employ support vector regression (SVR) [25] with our proposed features. In the case of linear functions, SVR finds parameters *w* and *b* for *f*(*x*) = 〈*w*, *x*〉+*b* by solving the following optimization problem:

(9) minimize 12||w||2+C∑i(ξi+ξi′),subject to yi−〈w,xi〉−b≤ϵ+ξi,yi−〈w,xi〉−b≥−ϵ−ξi′,ξi≥0,  ξi′≥0,

where *C* and *ϵ* are positive constants and (*x*_*i*_, *y*_*i*_) is a training data. Here, the penalty is added only if the difference between *f*(*x*_*i*_) and *y*_*i*_ is larger than *ϵ*. In our problem, *x*_*i*_ means a protein pair, and *y*_*i*_ means the corresponding interaction strength.

### 2.10. Relevance Vector Machine (RVM)

In this paper, we also employ relevance vector machine (RVM) [26] to predict strengths of PPIs. RVM is a sparse Bayesian model utilizing the same data-dependent kernel basis as the SVM. Its framework is almost the same as typical Bayesian linear regression. Given a training data {*x*_*i*_, *y*_*i*_}_*i*=0_^*N*^, the conditional probability of *y* given *x* is modeled as

(10) $$\begin{array}{c} p\left(y\;|\;x,w,\beta \right)=N\left(y\;|\;w^{T}\phi \left(x\right),\beta^{-1}\right), \end{array}$$

where *β* = *σ* ^2^ is noise parameter and *ϕ*(·) is a typically nonlinear projection of input features. To obtain sparse solutions, in RVM framework, a prior weight distribution is modified so that a different variance parameter is assigned for each weight as

(11) $$\begin{array}{c} p\left(w\;|\;\alpha \right)=\underset{i=0}{\prod }N\left(w_{i}\;|\;0,\alpha_{i}^{-1}\right), \end{array}$$

where *M* = *N* + 1 and *α* = (*α*_1_,…, *α*_*M*_)^*T*^ is a hyperparameter. RVM finds hyperparameter *α* by maximizing the marginal likelihood *p*(*y*∣*x*, *α*) via “evidence approximation.” In the process of maximizing evidence, some *α*_*i*_ approach infinity and the corresponding *w*_*i*_ become zero. Thus, the basis function corresponding with these parameters can be removed, and it leads sparse models. In many cases, RVM performs better than SVM especially in regression problems.

## 3. Results and Discussion

### 3.1. Computational Experiments

To evaluate our proposed method, we conducted computational experiments and compared with the existing method, APM.

### 3.2. Data and Implementation

It is difficult to directly measure actual strengths of PPIs for many protein pairs by biological and physical experiments. Hence, we used WI-PHI dataset with 50000 protein pairs [23]. For each PPI, WI-PHI contains a weight that is considered to represent some reliability of the PPI and is calculated from several different kinds of PPI datasets in some statistical manner to rank physical protein interactions. As strengths of PPIs, we used the value dividing the weight of PPI by the maximum weight for WI-PHI. We used dataset file “uniprot_sprot_fungi.dat.gz” downloaded from UniProt database [27] to get amino acid sequences, information of domain compositions, and domain regions in proteins. In this experiment, we used 1387 protein pairs that could be extracted from WI-PHI dataset with complete domain sequence via UniProt dataset. The extracted dataset contains 758 proteins and 327 domains. Since this dataset does not include protein pairs with interaction strength 0, we randomly selected 100 protein pairs that do not have any weights in the dataset and added them as protein pairs with strength 0. Thus, totally 1487 protein pairs were used in this experiment. We used “kernlab” package [28] for executing support vector regression and relevance vector machine and used the Laplacian kernel *K*(*x*, *y*) = exp⁡(−*σ*||*x* − *y*||). The dataset and the source code implemented by R are available upon request.

To evaluate prediction accuracy, we calculated the root mean square error (RMSE) for each prediction. RMSE is a measure of differences between predicted values ${\hat{y}}_{i}$ and actually observed values *y*_*i*_ and is defined by

(12) $$\begin{array}{c} \text{RMSE}=\sqrt{\frac{1}{N}\overset{N}{\underset{i=1}{\sum }}{\left({\hat{y}}_{i}-y_{i}\right)}^{2}}, \end{array}$$

where *N* is the number of test data.

### 3.3. Results of Computational Experiments

We preformed threefold cross-validation, calculated the average RMSE, and compared with APM [17]. For APM method, strengths of PPIs are inferred based on APM scores for domain pairs that consist of target proteins. However it is not always possible to calculate APM scores for all domain pairs from training set. Therefore, as test set, we used only protein pairs that consist of domain pairs with APM scores calculated via training set. (In all cases, about 40% of protein pairs in test set were used.) For the Laplacian kernel employed in both SVR and RVM, we selected kernel parameter *σ* by fivefold cross-validation from candidate set *σ* ∈ {0.01,0.02,…, 0.1}. The parameter *C* for the regularization term in the Lagrange formulation is set to *C* = 1,2, 5. Additionally, APM scores for each protein pair also can be used as input features. Therefore we also used APM scores as inputs for SVR and RVM and compared the model using APM scores with the model using our proposed features to confirm the usefulness of feature representation. Here, we used candidate set *σ* ∈ {3.0,3.1,3.2,…, 9.0} for kernel parameter *σ* of RVM + APM model because the model could not be trained with *σ* values smaller than 3. On the other hand, for *σ* of SVM + APM model, we used the same set as other models.

Table 1 shows the results of the average RMSE by SVR and RVM with our proposed features (DN and SPD of *k* = 1,2) and APM score and by APM, for training and test datasets. For training set, the average RMSEs by RVM with SPD of *k* = 2 were smaller than those by APM and others. Moreover, for test set, all the average RMSEs by RVM with SPD and DN were smaller than those by APM. The results suggested that supervised regression methods, SVR and RVM, with domain based features are useful for prediction of PPI strengths. Taking all results together, the model by RVM with SPD of *k* = 2 was regarded as the best for prediction of PPI strengths.

Since the average RMSEs of SVR with APM for both training and test dataset were smaller than those of original APM, SVR has potential to improve prediction accuracies. By contrast, the average RMSEs of RVM with APM became larger than those of original APM, and all average RMSEs of the models with APM for test set were larger than those of the models with DN and SPD. Accordingly, the results suggested that prediction accuracies were enhanced by feature representation and SPD is especially useful among these feature representations for predicting strengths of PPIs. Although DN and SPD of *k* = 1 have 654 and 42 dimensions for each protein pair, respectively, the average RMSEs with SPD of *k* = 1 for training set were smaller than those with DN. It implies that information of amino acid sequence in domain regions is more informative comparing with information of domain compositions to make a model fit in with dataset.

In contrast, the RMSEs by SVR with DN were smaller than those by others in some cases of test set.  Table 2 shows the numbers of relevance vectors and support vectors and the *σ* values selected by fivefold cross-validation in all cases. For the models with DN and APM scores, the numbers of relevance vectors were smaller than the numbers of support vectors. On the other hand, the numbers of relevance vectors were larger than the numbers of support vectors for the models with SPD feature in spite of the fact that usually RVM provides a sparse model compared with SVR. In RVM framework, sparsity of model is caused by distributions of each weight; that is, the number of relevance vectors is influenced by values and variances of each dimension of features rather than by the number of dimensions of features. Actually, each dimension of SPD feature almost always has widely varying values. In contrast, DN feature has many zeros, and APM score is inferred from training dataset and thereby has similar distribution. Thus, it is considered that many weights corresponding to features in RVM model did not become zero and the RVM models with SPD feature tended to be complex and to overfit the training data.

## 4. Conclusions

For the prediction of strengths of PPIs, we proposed feature space mappings DN and SPD. DN is based on the number of domains in a protein. SPD is based on the spectrum kernel and defined using the amino acid subsequences in domain regions. In this work, we employed support vector regression (SVR) and relevance vector machine (RVM) with the Laplacian kernel and conducted threefold cross-validation using WI-PHI dataset. For both training and test dataset, the average RMSEs by RVM with SPD feature were smaller than those by APM. The results showed that machine learning methods with domain information outperformed existing association method that is based on the probabilistic model of PPIs and implied that the information of amino acid sequence is useful for prediction comparing with only information of domain compositions. However, the models with SPD feature tended to be complex and overfitted to the training data. Therefore, to further enhance the prediction accuracy, improving kernel functions combining physical characteristics of domains and amino acids might be helpful.

## Figures and Tables

**Table 1:** Results of average RMSE for training and test data.

	*C* = 1		*C* = 2		*C* = 5	
	Training	Test	Training	Test	Training	Test
SVR + DN	0.10472	0.12573	0.10656	0.12600	0.09982	** 0.12484**
RVM + DN	0.09210	0.12873	0.09178	0.12881	0.09474	0.12908
SVR + SPD (*k* = 1)	0.08819	0.12699	0.08080	0.12954	0.07927	0.12903
RVM + SPD (*k* = 1)	0.02848	0.12743	** 0.01504**	0.12706	0.03276	0.12792
SVR + SPD (*k* = 2)	0.08891	0.12654	0.08188	0.12782	0.08117	0.12909
RVM + SPD (*k* = 2)	** 0.02529**	** 0.12470**	0.02301	** 0.12476**	** 0.02243**	0.12493
SVR + APM	0.06846	0.13112	0.06795	0.13247	0.06791	0.13277
RVM + APM	0.07052	0.13556	0.07037	0.13550	0.07032	0.13493

APM	Training = 0.06811, Test = 0.13517					

**Table 2:** The number of relevance vectors (RVs) and support vectors (SVs) for each model with DN, SPD, and APM and the selected *σ* values for each fold.

		*C* = 1		*C* = 2		*C* = 5	
		SVR	RVM	SVR	RVM	SVR	RVM
		SVs (*σ* value)	RVs (*σ* value)	SVs (*σ* value)	RVs (*σ* value)	SVs (*σ* value)	RVs (*σ* value)
Fold 1	DN	271 (0.02)	113 (0.05)	271 (0.01)	123 (0.07)	308 (0.01)	74 (0.02)
	SPD (*k* = 1)	367 (0.01)	448 (0.02)	402 (0.02)	680 (0.05)	402 (0.02)	537 (0.03)
	SPD (*k* = 2)	392 (0.01)	502 (0.03)	409 (0.01)	628 (0.05)	421 (0.01)	628 (0.05)
	APM	362 (0.08)	4 (5.00)	361 (0.10)	6 (4.80)	357 (0.04)	6 (5.80)

Fold 2	DN	280 (0.02)	94 (0.08)	281 (0.01)	92 (0.09)	314 (0.01)	82 (0.04)
	SPD (*k* = 1)	408 (0.01)	617 (0.04)	453 (0.04)	706 (0.06)	411 (0.01)	545 (0.03)
	SPD (*k* = 2)	430 (0.01)	558 (0.04)	435 (0.01)	618 (0.05)	495 (0.04)	654 (0.06)
	APM	375 (0.10)	5 (6.50)	372 (0.10)	6 (6.90)	373 (0.04)	4 (5.50)

Fold 3	DN	321 (0.04)	107 (0.08)	289 (0.01)	107 (0.10)	330 (0.01)	107 (0.08)
	SPD (*k* = 1)	371 (0.01)	439 (0.02)	412 (0.03)	658 (0.05)	382 (0.01)	305 (0.01)
	SPD (*k* = 2)	387 (0.01)	625 (0.06)	418 (0.02)	529 (0.04)	398 (0.01)	529 (0.04)
	APM	368 (0.08)	3 (7.10)	368 (0.04)	3 (6.70)	372 (0.01)	5 (4.20)
